# Supplementary material for: Yuzhoua juvenilis: Another Angiosperm Seen in the Early Permian?
Source: Life (Basel). 2025 Feb 12;15(2):286. doi: 10.3390/life15020286 (PMC11856813; doi:10.3390/life15020286)
Supplement: Supplementary file 1 [file life-15-00286-s001.zip › life-3441966-supplementary.pdf]

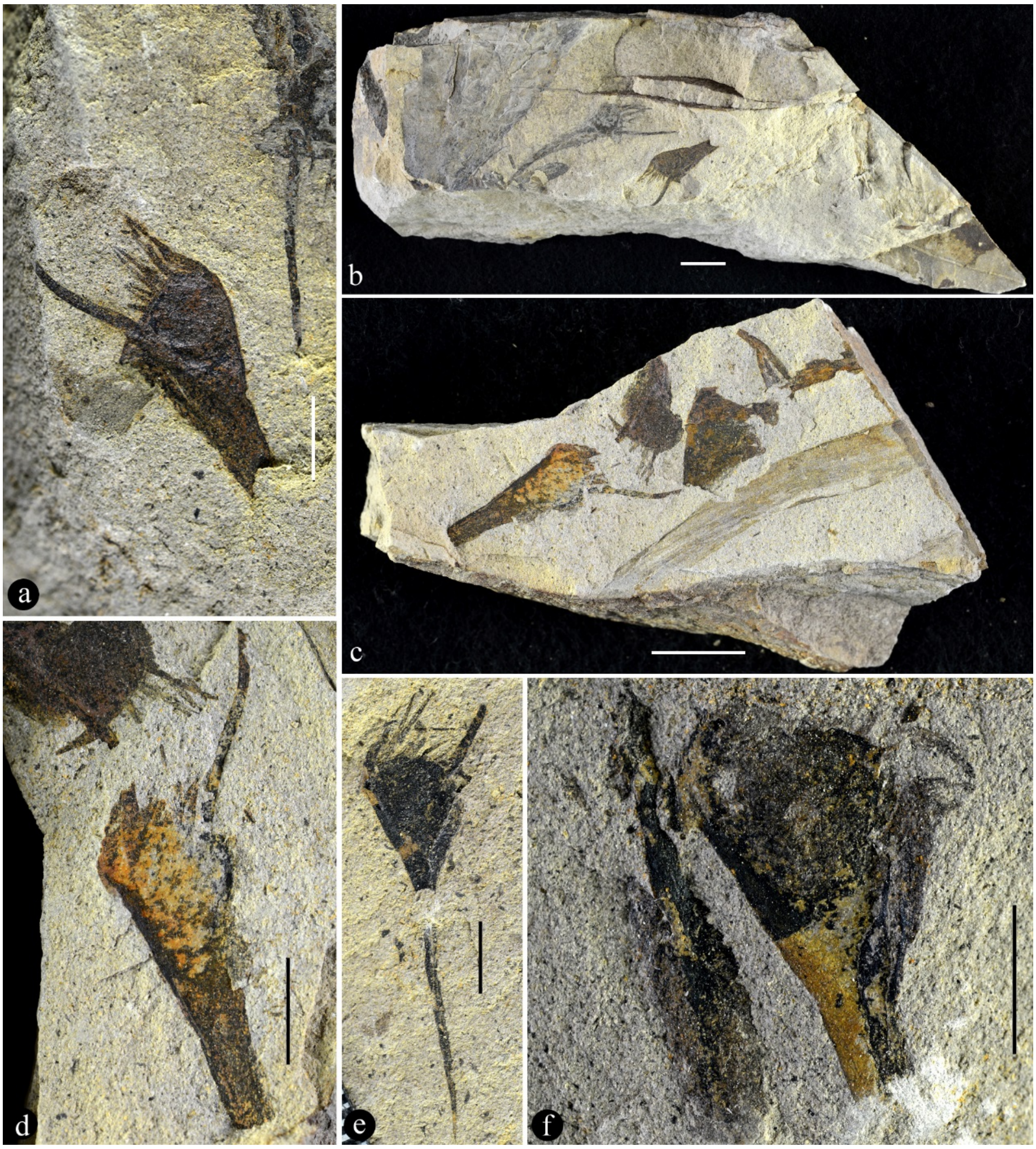

Figure S1. Six fruits of *Yuzhoua* gen. et sp. nov. A. Holotype, one of two fruits on the same specimen shown in Fig. S1b. It is still partially covered by the sediments. PB205390a. Scale bar = 5 mm. **B.** Two fruits on the same specimen**.** PB205390. Scale bar = 10 mm. **C.** There are at least two fruits on this specimen. Paratype, 41HIV0181. Scale bar = 10 mm. **D.** One of two fruits in Fig. S1c. 41HIV0181a. Scale bar = 5 mm. **E.** Another fruit. Paratype, PB205391**.** Scale bar = 5 mm. **F.** Another fruit. Paratype, 41HIV0182. Scale bar = 5 mm.
